# Regional Variation in Pig Farmer Awareness and Actions Regarding Japanese Encephalitis in Nepal: Implications for Public Health Education

**DOI:** 10.1371/journal.pone.0085399

**Published:** 2014-01-09

**Authors:** Santosh Dhakal, Durga Datt Joshi, Anita Ale, Minu Sharma, Meena Dahal, Yogendra Shah, Dhan Kumar Pant, Craig Stephen

**Affiliations:** 1 National Zoonoses and Food Hygiene Research Centre, Kathmandu, Nepal; 2 Centre for Coastal Health, Nanaimo British Columbia, Canada and Faculty of Veterinary Medicine and Faculty of Medicine, University of Calgary, Calgary, Alberta, Canada; Institut National de la Recherche Agronomique, France

## Abstract

Japanese encephalitis (JE) is a mosquito-borne zoonotic disease that has pigs as the major amplifying hosts. It is the most important cause of viral encephalitis in people in Nepal and is spreading in its geographic distribution in that country. Pig farming is increasing in Nepal due to reducing cultural biases against pigs and government programs to support pig farming for poverty alleviation. Major strategies for JE prevention and control include education, vector control, and immunization of people and pigs. This study used a survey of 400 pig farmers in 4 areas of Nepal with different JE and pig farming histories to explore regional variations in farmer awareness and actions towards JE, the association of awareness and actions with farm and farmer variables, and the implications of these associations for public health education. Exposure to JE risk factors was common across pig farms and pig farming districts but there were significant district level differences in knowledge and practices related to on-farm JE risk reduction. Social factors such as literacy, gender, and cultural practices were associated with farmer attitudes, knowledge and practices for JE control. JE vaccine uptake was almost non-existent and mosquito control steps were inconsistently applied across all 4 districts. Income was not a determining factor of the differences, but all farmers were very poor. The low uptake of vaccine and lack of infrastructure or financial capacity to house pigs indoors or away from people suggest that farmer personal protection should be a priority target for education in Nepal. This study re-enforces the need to attack root causes of people’s personal disease prevention behaviours and take into account local variation in needs and capacities when designing health or agriculture education programs.

## Introduction

Japanese encephalitis (JE) is the most important viral encephalitis in Nepal and Asia [1], [2]. It is a mosquito borne disease caused by a flavivirus that cycles between birds, pigs and people [3]. Its distribution has, in recent years expanded and is expected to spread more widely geographically with anticipated changes in climate, land use (particularly rice and pig farming and urbanization), and access to health care [4], [5]. The high case fatality rate, high rate of severe long lasting neurological symptoms and the majority of deaths occurring in children make JE a major public health problem [6]. JE is not homogenously distributed in Nepal. The disease was first confirmed in western Nepal in 1978 [7] in the lowland plains of Terai which borders India, and has regularly occurred since then with epidemic peaks every 2–5 years [8]. JE has now been documented in 54 of the 75 districts of Nepal, including 24 hill and mountain districts, and is considered to be endemic in the Kathmandu Valley as well as in 24 other districts [8], [9], [10].

The World Health Organization Regional Office for South-East Asia (SEARO/WHO) has pointed out four major strategies for JE prevention and control; health education, vector control, immunization of people and pigs, and epidemic preparedness and response [12]. Other countries like Japan, South Korea and Taiwan have successfully controlled JE by human and pig vaccination, modernization of pig farms, change in agricultural practices and improved living standards [4], [11]. The investments required to achieve these changes is beyond the current economic capacity of Nepal. Even regular human vaccination is not affordable or sustainable at the present time. Low-cost alternatives need to be found to begin to address the SEARO/WHO recommendations.

Pig farming is increasing in Nepal due to reduced cultural biases against pigs and new government programs to support pig farming as a low cost means to create income for poverty alleviation and to improve food security. A 48% increase in the Nepal pig population in 14 years [13] shows how fast this industry is growing. Pig farms are not homogeneously distributed in Nepal. Pig farming is most prevalent in the eastern and central regions of the country where the pig-raising ethnic community predominates and is also more common in peri-urban areas because of an increased demand of pig meat in urban areas. Because pigs are a major JE virus amplifying hosts [14], ensuring pig farmers are aware of and able to use means for personal and community risk reduction should be an important part of a Nepalese JE prevention strategy. Our previous study in the Kathmandu district [15] found that the pig farmers had high occupational exposures to JE risk factors but they also had low levels of awareness and adoption of preventive measures against JE. Knowledge, beliefs and practices can affect how individuals or groups apply control efforts against disease [16], [17]. Because we found socio-economic associations with knowledge about JE and the use of preventive practices in the Kathmandu district, in this study we sought to replicate Dhakal et. al. [15] in four districts with different experiences with both JE and pig farming to see if there were regional variations that may influence how farmer JE education programs might need to be adapted to local needs and situations. In this study we used a survey methodology to ascertain information on what pig farmers knew about JE, its risk factors and/or its control in; a long term endemic region (>30yrs) that is the source of most human JE cases in the country (Rupandehi district); a neighbouring endemic district with lower numbers of outbreaks (Kapilvastu near the Indian border); an area of endemic JE with the highest level of pig amplifying hosts (Morang district); and a region of comparatively recent JE emergence in the hill region (Kathmandu valley).Our objective was to determine if there was regional variation in JE knowledge about the disease and practices within pig farm communities to help public health and agriculture extension planners tailor educational material to local needs.

## Methods

This research was conducted from September 2011 to June 2012 in four districts of Nepal as described above. Within each district 2–4 communities known for active pig farming were selected in order to recruit 100 participants per district. The same procedure used in Dhakal et. al., 2012 was used for all districts. One hundred farms were selected by simple random sampling based on a sampling frame derived from local agriculture agency registry of pig farms. Before visiting the farms, the survey team went to the community and introduced the project to the para-veterinarians servicing the area and to community leaders. Farmers were provided the incentive to participate by being offered free physical examinations of the pigs owned by the farmers and treatment of any ill animals by a veterinarian. Local para-veterinarians were informed about any health issues or animal treatments after each visit to ensure follow up. Farmers were also offered a short training session on JE and its preventive measures after they completed the survey. This recruitment process resulted in 100% compliance with all requests to participate.

### Ethics statement

Participation in the survey was voluntary and all farmers were informed of their right to withdraw at any time in the survey and were required to give their consent to participate. Verbal consent to participate was requested because the high anticipated rate of illiteracy would preclude many from providing written consent. Consent was noted by the field investigator. All responses were recorded anonymously and without any identifying information. Ethical approval was provided for this procedure, including the use of verbal consent, by the ethics committee of the National Zoonoses and Food Hygiene Research Centre (approval number NZFHRC 22/10/7/2010/2011).

Participating farmers were verbally asked questions from a standardized survey and their oral response recorded by the interviewer. There were six main parts to the questionnaire; (i) farmer attributes such as gender, education, income, experience and training, (ii) farm attributes such as the number of pigs raised, management, and source and marketing of pigs, (iii) farmer proximity to known risk factors such as rice fields, pig barns, and standing water, (iv) farmer knowledge about JE and what can be done to prevent the disease, (v) JE specific practices used by the farmer for themselves, their pigs and their family including vaccination and mosquito avoidance and (vi) information on clinically compatible signs or past diagnosis of JE in people or animals on the farm. As this project was focused on farmer knowledge and exposure to known risks, we did not undertake a sero-survey of people or animals to document the prevalence of JE, but instead relied on self-reported information.

The survey contained both open- and closed-ended questions. Open-ended questions were grouped based on their similarities and turned into closed-ended replies after a thematic review of the answers. All answers were coded and entered into the SPSS software version 19 (http://www-01.ibm.com/software/analytics/spss/). Descriptive statistics used for analysis included frequencies, sums, ranges and means, and the chi-squared test was used as a test of association with p< 0.05 selected as the level for statistical significance. Chi-square was estimated to identify association between JE knowledge and predictors; mosquitoes avoiding practice and predictors. Univariate analysis was undertaken to calculate the odds ratio with 95% confidence interval. Significant variables were entered in to logistical model. Logistic regression was used for multivariate analysis of potential predictors of farmer’s knowledge about JE; with p<0.05 used as a threshold for statistically significant interactions. The default “enter” method was used in SPSS for variable selection in which all variables in a block were entered in a single step.

## Results

All farmer houses in Kathmandu, Morang and Kapilvastu and all except one in Rupandehi were within 500 meters of the pig pens. Most pig farms were small family operations. The average number of pigs in Kathmandu was 26, followed by 8 in Kapilvastu, 6 in Rupandehi and 4 in Morang study area. Free range or open-air pens were common types of housing in all practices. Eighty-seven percent (346/400) of houses overall were within 1 km of rice paddy fields (range of 73–95%) and 91% (362/400) of farm houses within 1 Km of a standing water sources (range 80–99%) (table 1). There were regional differences in proximity to potential breeding sites for mosquitoes (paddy field [p<0.001]; standing water sources (p<0.001)]. All pig farmers had encountered mosquitoes on their farm and all reported mosquito biting their pigs and themselves. All farmers reported encountering wild birds on their farms. Among the 400 farmers, 62 (15.5%) also had domestic ducks and 193 (57%) had duck farms within 1 Km distance (ranging from 100%, 78%, 40% and 37% respectively, in Kathmandu, Morang, Kapilvastu and Rupandehi).

**Table 1:** Farmer and farm characteristics for 400 pig farmers and farms in 4 districts of Nepal.

Parameters	Kathmandu (n = 100)	Morang (n = 100)	Rupandehi (n = 100)	Kapilvastu (n = 100)	P value
Male: Female respondents	50:50	58:42	48:52	79:21	<0.001
Number of farmers self-declared as illiterate	39	55	23	78	<0.001
Farms with monthly income≤ 10000 NRS from pig farming	70	95	82	94	<0.001
Pig farming as sole occupation	73	18	27	25	<0.001
Number of farms who owned the land used for pig farming	15	65	90	87	<0.001
Number of farmers with <3 years’ experience pig farming	27	16	21	40	0.001
Farm houses located ≤ 500 m from the pig farm	100	100	99	100	NT
Farm houses located ≤ 1 km from rice field	95	88	73	90	<0.001
Farm houses located ≤ 1 km from standing water bodies	99	91	92	80	<0.001
Number of farmers who knew people can get diseases from pigs	72	39	67	12	<0.001
Number of farmers who heard about JE	42	25	38	15	<0.001
Number of farmers vaccinating pigs against at least one disease	87	13	44	6	<0.001

There were district level differences in the ratio of male:female respondents (p<0.001) mainly due to Kapilvastu, where females were generally reluctant to respond to interviewers from outside of their district. Across the four study districts we found significant differences in literacy rate (p<0.001), monthly income (p<0.001), pig farming being the sole income source (p<0.001), land ownership (p<0.001) and experience as a pig farmer (p = 0.001) (table 1). Out of 400 pig farmers 195 (49%) were illiterate and the monthly income of 85% of farm families was not more than 10,000 NRS. Pig farming was sole source of income for 73% of pig farmers in Kathmandu study area but overall only 36% (143/400) farmers had this as sole occupation. The other occupations however, were low income generating type. Seventy-four percent (296/400) of pig farmers had 3 or more years’ experience in pig farming. Many pig farms in Kathmandu were mobile due to being located on leasehold land but in other districts they were more permanent. Except for few farms in Rupandehi, all pig farms were of poor sanitary conditions and lacked biosecurity measures.

Out of 400 pig farmers, only 36 (9%) had received formal training on pig husbandry management or disease. Training was provided to these 36 individuals from government (n = 21,58%) followed by farmers groups (n = 9, 25%) and non-governmental organizations.

(n = 6, 17%). Of the 364 farmers who had not received training, 198 (54%) said they did not know training was available, 146 (40%) said they did not know where to go for training and 20 (6%) said they couldn’t afford training. Seventy-three percentages of pig farmers (292/400) said they learned about pig diseases through their own experience or through pig farming friends and the community. Only 27% (108/400) said they got information from veterinary sources. We found field veterinarians were the most trusted source of information on immunization practices for pigs in all districts: 83% of farmers relied on veterinarians followed by the pig farming community members (17%) for making immunization decisions.

Less than half (190/400) of the pig farmers interviewed knew they could acquire a disease from pigs but only 10% (40) could name a pig associated zoonosis. They named swine flu (17/40), JE (21/40), and neurocysticercosis (2/40). However, 30% (120/400) of the farmers were aware of JE. Eighteen percent (73/400) knew signs of JE in people, 7% (29/400) knew JE signs for pigs, 17% (66/400) knew JE was transmitted by mosquitoes, 9% (34/400) knew it can be prevented by vaccine in pigs and 15% (59/400) knew it is vaccine preventable in people. There was, however regional variation in the proportion of farmers who were aware of JE; Kathmandu 42%, Rupandehi 38%; Morang 25% and Kapilvastu 15%. Farmers who were aware of JE were more likely to know that people could acquire diseases from pigs (p<0.001) (table 1). Of the 120 farmers who were aware of JE, 53% learned about the disease through media sources, 28% from friends and community members, 9% from health care providers; 7% from training events and 4% through academic study. There was a significant difference in where farmers in different districts learned about JE (p = 0.008). For example, 20% (20/100) of farmers in Rupandehi heard about JE through the media compared to only 8% (8/100) in Kalivastu and 20% (20/100) of farmers in Katmandu heard about JE from friends or the community compared to 2% (2/100) in Morang.

Only one family in Rupandehi from our survey group reported JE being diagnosed in a family member. Reports of clinically compatible signs like high fever, unconsciousness, severe headache, neck rigidity, convulsion and/or paralysis were infrequently reported in farm family members (table 2). Severe headache were more commonly reported in Kapilvastu families (p<0.001). There were significant differences between districts for JE compatible signs in in the pig health including; abortion (p<0.001), weak piglets (p<0.001) and convulsion (p<0.001).

**Table 2:** Pig and human clinical signs compatible with Japanese Encephalitis as reported by 400 pig farmers in four districts of Nepal.

	Kathmandu (n = 100)	Morang (n = 100)	Rupandehi (n = 100)	Kapilvastu (n = 100)	P-Value
Pig Health Disorders (n)					
Abortion	36	8	10	15	<0.001
False Pregnancy	12	5	6	7	0.24
Weak piglets	36	5	15	66	<0.001
Convulsions	20	5	7	3	<0.001
Hydrocephalus	2	1	2	1	NT
Swollen testicles	2	1	0	2	NT
Human Health Disorders (n)					
High fever	8	14	17	21	0.07
Severe headache	12	7	7	46	<0.001
Unconsciousness	2	3	1	2	NT
Neck rigidity	1	1	0	0	NT
Convulsion	1	2	1	0	NT
Paralysis	0	2	2	0	NT

NT indicates associations were not tested because of the magnitude of difference.

Education status of the farmers, study district, farmer’s sex and whether or not the farmer had raised pigs for more than 3 years were each found to be significantly associated with whether or not the farmer was aware of JE (table 3). Only one farmer reported that family members had received JE vaccine. This same family also reported the death of a family member due to JE and explained this was the motivation to have the rest of the family immunized. Nearly 38% (150/400) had vaccinated their pigs against various diseases (120 against classical swine fever, 3 against foot and mouth disease, 25 against both classical swine fever and foot and mouth disease, and 2 against hemorrhagic septicemia). Vaccines against JE were not commercially available for pigs in Nepal. There was a significant difference in pig vaccination status in four districts for other pig diseases (p<0.001): 87% of pig farmers in Kathmandu had vaccinated pigs against at least one disease followed by 44 in Rupandehi, 13% in Morang and only 6% in Kapilvastu (table 1). Reasons for not vaccinating pigs varied and included; they didn’t know pigs needed vaccine (117/250), they didn’t have problems in pigs that needed vaccines (94/250), they couldn’t afford to purchase vaccines (13/250) or they didn’t think vaccines worked (3/250). The remaining 23/250 couldn’t say why they were not vaccinating pigs.

**Table 3:** Associations between farmer attributes and practices and awareness of Japanese Encephalitis (JE) among Nepalese pig farmers.

Association tested	P value	Odds ratio	95%CI
Literacy and awareness of JE	<0.001	3.62	(2.27–5.76)
Know people can get disease from pigs and awareness of JE	<0.001	4.29	(2.61–7.05)
Gender and awareness of JE	0.004	1.89	(1.20–2.97)
Income ≤ 10000 NRS and attendance at pig farming training sessions	<0.001	5.19	(2.49–10.82)
Literacy and use of at least one mosquito avoiding practices	0.01	4.41	(1.22–15.89)
Time period of raising pig and awareness of JE	0.02	1.69	(1.06–2.70)
Awareness of JE and use of at least one mosquito avoiding practices	0.03	6.26	(0.87–44.99)
Attendance at pig farming training sessions and awareness of JE	0.08	1.76	(0.88–3.52)
Income ≤ 10000 NRS and awareness of JE	0.12	0.68	(0.38–1.20)
Attendance at pig farming training sessions and use of at least one mosquito avoiding practices	0.24	3.24	(0.20–53.20)
Income ≤ 10000 NRS and use of at least one mosquito avoiding practices	0.32	0.40	(0.05–3.07)

Ninety-six percent (385/400) of the farmers knew at least one method of preventing mosquito bites in their family. The remaining 4% (15/400) were unaware of means to prevent or avoid mosquito bites. The various techniques used included use of window screen, use of repellents, use of mosquito coils, staying indoor at dawn/dusk, wearing clothes that cover full body, improving drainage and use of mosquito nets (table 4). There were significant differences across districts in the use of various mosquito avoiding techniques (table 4). There was also variation in the frequency or intensity of their use. For example, reporting use of mosquito nets could mean that several family members used nets or only the children used them. Twenty-four percent (97/400) of the farmers reported they practiced mosquito avoidance practices in their pig sheds. The practices included spraying chemicals (47/97), maintaining cleanliness (7/97), using smoke from a fire (42/97) and using repellents (1/97). There was a significant association between whether or not a person was aware of JE and whether or not they practiced at least one mosquito avoiding practices (p = 0.03). There was also an association between whether or not a person was literate and the use of mosquito bite prevention technique (p = 0.01). However, there was no association between the use of at least one mosquito bite prevention technique and training on pig farming (p = 0.24) or household income (p = 0.32) (table 3).

**Table 4:** Use of mosquito avoiding practices by 400 pig farmers in four different districts in Nepal.

	Kathmandu (n = 100)	Morang (n = 100)	Rupandehi (n = 100)	Kapilvastu (n = 100)	p value
Use window screen	11	8	42	6	<0.001
Use repellants	25	8	18	4	<0.001
Use mosquito net	41	51	88	38	<0.001
Improve drainage	38	71	65	6	<0.001
Use mosquito coil	69	49	68	50	0.001
Stay indoors at dawn/dusk	39	22	42	42	0.007
Wear clothes that fully covers the body	40	22	32	4	<0.001

NT indicates associations were not tested because of the magnitude of difference.

Logistic regression failed to produce model with significant predictive value (Cox and Snell R^2^  =  0.197). A final model had five variables literacy, gender, time period of raising pigs, mosquitoes avoiding practices and knowledge about pig disease associated with awareness of JE. All of these variables were significant in the final model except mosquitoes practice (Wald = 2.06; p = 0.151) (table 5).

**Table 5:** Final logistic regression model farmer knowledge of Japanese Encephalitis and predictors (Cox and Snell; R^2^  =  0.197).

Variables	B	SE	Wald	Sig	Exp (B)
Constant	–0.248	0.311	0.634	0.426	0.781
Literacy	–0.639	0.275	5.39	0.020	0.528
Gender	0.773	0.263	8.64	0.003	2.166
Time period of pig raining	0.738	0.278	7.076	0.008	2.09
Mosquitoes avoiding practice	–0.416	0.289	2.06	0.151	0.660
Knowledge about pig disease	–1.608	0.287	31.336	0.000	0.200

## Discussion

Each of the four study districts had recent histories of JE. In the 5 year period from 2007 to 2011, there were 157 JE human cases reported in Kathmandu, 89 cases reported in Morang, 16 cases reported in Rupandehi and 16 cases reported in Kapilvastu district [18]. JE risk factors were common across farms and farm families in all four districts but there were district level differences in knowledge and practices related to on-farm JE risk reduction. A previous survey in Nepal identified three JE control priorities;(i) information, education and communication strengthening to increase awareness of individuals and communities;(ii) behavioral changes to increase prevention practices and (iii) environmental interventions to reduce risk factors [19]. We found that Kapilvastu district lagged behind in all three of these priority areas followed by the Morang district. Farmers in Kapilivastu not only had the lowest level of awareness of JE, but also had the lowest proportion of farmers who were (i) literate, (ii) had more than 3 years pig farming experience, (iii) had higher incomes, (iv) knew that people could acquire diseases from pigs and (v) used methods for mosquito bite avoidance. In all regions, women were less likely to have heard of JE than men. Literacy rate may be an important cause of these differences. Districts where farmers had a higher level of literacy (Kathmandu 61% and Rupandehi77%) had a higher rate of JE awareness than the districts with lower farmer literacy rates (Morang 45% and Kapilvastu 22%). The overall literacy rate in our sample population (51%) was lower than the national literacy rate of 65.9%. The literacy rate for women is less (57.4%) than that of men (75.1%) in Nepal [20]. Efforts to change farmer JE prevention behaviours will need to take into account the implications of low literacy rates when designing education programs.

Mosquito control would seem a critical target for Nepal not only due to the inconsistent use of control measures found in this study but also due to the presence of multiple vector-borne diseases in Nepal such as malaria and dengue fever. Community-based educational interventions have been shown elsewhere to affect understanding and involvement in mosquito control and vectorborne disease prevention [21], [22]. Work on community education for vector control to eliminate lymphatic filariasis in southern India, using pre-post surveys in exposed and control villages, found that an 87% reduction in mosquito density could be achieved for a per capita cost of $0.32 [23]. Programs targeting mosquito control have resulted in declines in JE elsewhere. For example, in Assam, India, a sharp reduction in JE sero-conversion rates in people and pigs was achieved when insecticide treated nets were used in both people and pigs [24]. Similarly, a population based case-control study in China found that use of insecticide treated nets was associated with significant reduction in JE cases [25].There were significant differences in use of various mosquito control techniques in different districts in our study, often related to socio-economic factors. For example, the use of window screen was higher in Rupandehi because the pig farmers more often had permanent houses with windows compared to pig farmers of Kathmandu, Morang and Kapilvastu districts who often lived in homes without windows. Higher literacy rates (p = 0.01) and being aware of JE (p = 0.03) were associated with the use of at least 1 mosquito control practice but income (p = 0.32) and training on pig farming (p = 0.24) were not.

A number of countries have achieved tremendous reductions in the number of human JE cases by vaccination of pigs and people along with environmental changes like separation of houses and pigpens [26]. Although the Nepal government had JE vaccination programs for people in our study districts, there was virtually no uptake in the pig farming community. Only one family had been vaccinated and this was the same family where JE had taken the life of a family member. Pig farmers were not opposed to immunization as they had vaccinated their pigs for diseases other than JE and supplemental questions founds that many had their children vaccinated for standard childhood vaccine preventable diseases. Reasons for lack of pig vaccination may be as easy to explain because of the lack of readily available commercial vaccine and lack of perception that JE caused illness in the pigs. Data on reasons for the exceedingly low uptake of JE vaccine in Nepalese pig farming families are lacking. A survey of mothers on demand side barriers to childhood vaccination in the Terai area of Nepal found lack of knowledge, misconception about immunization, lack of access to health services, heavy household work related to poverty, lack of permission from family to visit a health facility and religion, caste and gender factors were associated with lower vaccine uptake [27]. Public concern over adverse reactions led to refusal of the JE vaccination and a consequent decrease in coverage rate in Korea [28]. Further study is required to determine why pig farmers, who appear to be a high risk occupation group, have such low levels of vaccine uptake in a country where JE vaccine is free for people.

Income was not found to be a significant predictor of JE knowledge or practices. Although there were statistical differences in the proportion of homes with incomes more than 10000 NRS per month, all of the farm household could be considered to be very poor and well below the national average income of 202,374 NRS per year [29]. There is evidence for other vector-borne diseases that socio-economic status affects the uptake of prevention and treatment interventions [30]. It may be possible that the study population was so far below an income threshold that it obscured the effect of household income on JE prevention and control. There was a significant association between income and having had training on pig farming. It is not clear if the training increased income or income provided funds to attend training sessions. Having training or not had no bearing on awareness of JE likely because training was focused on pig husbandry and management rather than zoonotic disease prevention and control. Seventy three percent of pig farmers learned about pig diseases through their own experiences or from other pig farmers whom they trusted, suggesting that social networks may be key to disseminating information on JE. Field veterinarians were also a trusted source, especially about immunization and are therefore another key conduit of information into the community. Except for Kathmandu where the pig farming community was a major source of information, media was the main source of information on JE and thus should be targeted for public education campaigns. Media campaigns will need to use means for knowledge mobilization that can target the proportion of farmers and farm household members who are illiterate as well as be sensitive to gender differences in use of media sources.

There were three key reasons to prioritize pig farming families for JE education and control. First, this study showed that this occupational group lived and worked in close proximity to key JE risk factors like pigs, rice fields, ducks, wild birds, mosquitoes and standing water. The major JE vector *Culex tritaeniorhynchus* breeds predominantly in rice fields and open sunlit temporary and permanent habitats with vegetation and they have average flight range of 1.5 km [31]. We found the pig farms in all study areas were located within this flight range from rice fields and the standing water sources. Second, there was a low rate of use of JE prevention for families or pigs. Third, pig production is increasing in Nepal and is expected to grow. Add to these factors the spectre of an expanding JE range associated with climate change and land use changes and there is ample reason to conclude that pig farming families should be priority targets of JE control campaigns. Validation of this assumption will require case-control or similar studies to determine if pig farmers are indeed at a higher risk for JE disease in Nepal.

The design and delivery of future pig farm families JE education on prevention and control will need to take into consideration, not only how pig farmers differ from other members of society in terms of their exposure risks and capacities to understand, access and apply JE control actions but also how to tailor programs to differences in socio-economic variables across districts. This study re-enforces the need to attack root causes of people’s personal disease prevention behaviours, such as literacy, when aiming to have wide impacts from public health or agriculture extension and education programs.
